# Dabrafenib and trametinib therapy in an elderly patient with non‐small cell lung cancer harboring the *BRAF* V600E mutation

**DOI:** 10.1111/1759-7714.13756

**Published:** 2020-11-20

**Authors:** Yosuke Dotsu, Minoru Fukuda, Noritaka Honda, Hiroshi Gyotoku, Yoshihisa Kohno, Takayuki Suyama, Yasuhiro Umeyama, Hirokazu Taniguchi, Shinnosuke Takemoto, Hiroyuki Yamaguchi, Taiga Miyazaki, Noriho Sakamoto, Yasushi Obase, Hiroaki Ikeda, Kazuto Ashizawa, Hiroshi Mukae

**Affiliations:** ^1^ Department of Respiratory Medicine Nagasaki University Graduate School of Biomedical Sciences Nagasaki Japan; ^2^ Clinical Oncology Center Nagasaki University Hospital Nagasaki Japan; ^3^ Department of Rehabilitation Medicine Hamamatsu City Rehabilitation Hospital Hamamatsu Japan; ^4^ Molecular Pharmacology Program and Department of Medicine Memorial Sloan Kettering Cancer Center New York New York USA; ^5^ Department of Oncology Nagasaki University Graduate School of Biomedical Science Nagasaki Japan

**Keywords:** *BRAF* V600E, dabrafenib, elderly, non‐small cell lung cancer, trametinib

## Abstract

Dabrafenib and trametinib therapy for *BRAF* V600E‐mutant non‐small cell lung cancer (NSCLC) has demonstrated strong antitumor effects in clinical trials and has been approved for use in clinical practice. However, the efficacy and safety of this combination therapy in elderly patients remain unclear. An 86‐year‐old male patient, who had been diagnosed with lung adenocarcinoma with the *BRAF* V600E mutation, received dabrafenib and trametinib combination chemotherapy. The tumor shrunk rapidly; however, therapy was discontinued after 40 days because adverse events (hypoalbuminemia, peripheral edema, and pneumonia) developed. Although this targeted combination therapy seemed to cause relatively severe adverse events compared with single‐agent targeted therapy in this “oldest old” elderly patient, the marked tumor shrinkage prolonged the patient's life and helped him to maintain a good general condition. Active targeted therapy may therefore be considered with appropriate drug dose reduction instead of conservative treatment, even if a patient is extremely old.

## Graphical abstract

An 86‐year‐old male patient, who had been diagnosed with lung adenocarcinoma with BRAF V600E mutation, received dabrafenib and trametinib combination chemotherapy. The tumor shrunk rapidly; however, therapy was discontinued after 40 days because adverse events (hypoalbuminemia, peripheral edema, and pneumonia) developed. Although this targeted combination therapy seemed to cause relatively severe adverse events compared with single‐agent targeted therapy in this “oldest old” elderly patient, the marked tumor shrinkage prolonged the patient's life and helped him to maintain a good general condition.

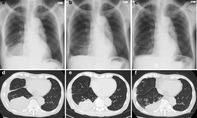

## Introduction

The v‐raf murine sarcoma viral oncogene homolog B1 (*BRAF*) gene has been found to function as a driver oncogene through mitogen‐activated protein kinase (MAPK) signaling^1^ in patients with non‐small cell lung cancer (NSCLC). *BRAF* mutations occur in approximately 1%–4% of cases of NSCLC, and the most common *BRAF* mutation is V600E, which results in glutamate being substituted for valine at codon 600.^1^, ^2^ The efficacy of dual BRAF and meiotic chromosome‐axis‐associated kinase (MEK) inhibitors has been evaluated in previously treated and untreated patients with *BRAF* V600E‐mutant metastatic NSCLC, in whom it achieved overall response rates of 63%–64% and complete response rates of 4%–6%.^3^, ^4^ However, the ages of the subjects of these studies ranged from 58–71 and from 62–74 years, respectively, and the efficacy and safety of combination therapy for such disease in elderly patients remain unclear. Here, we describe a case, in which an elderly (“oldest old”) patient with *BRAF* V600E‐mutant NSCLC adenocarcinoma was treated with dabrafenib and trametinib. Although the treatment brought durable tumor shrinkage and symptom relief, it had to be terminated due to the occurrence of marked hypoalbuminemia and edema. Informed consent from the patient and the permission of ethics committee of Nagasaki University Hospital (Permission Number 20081733) were obtained to publish the report and accompanying images.

## Case report

**Figure 1:**
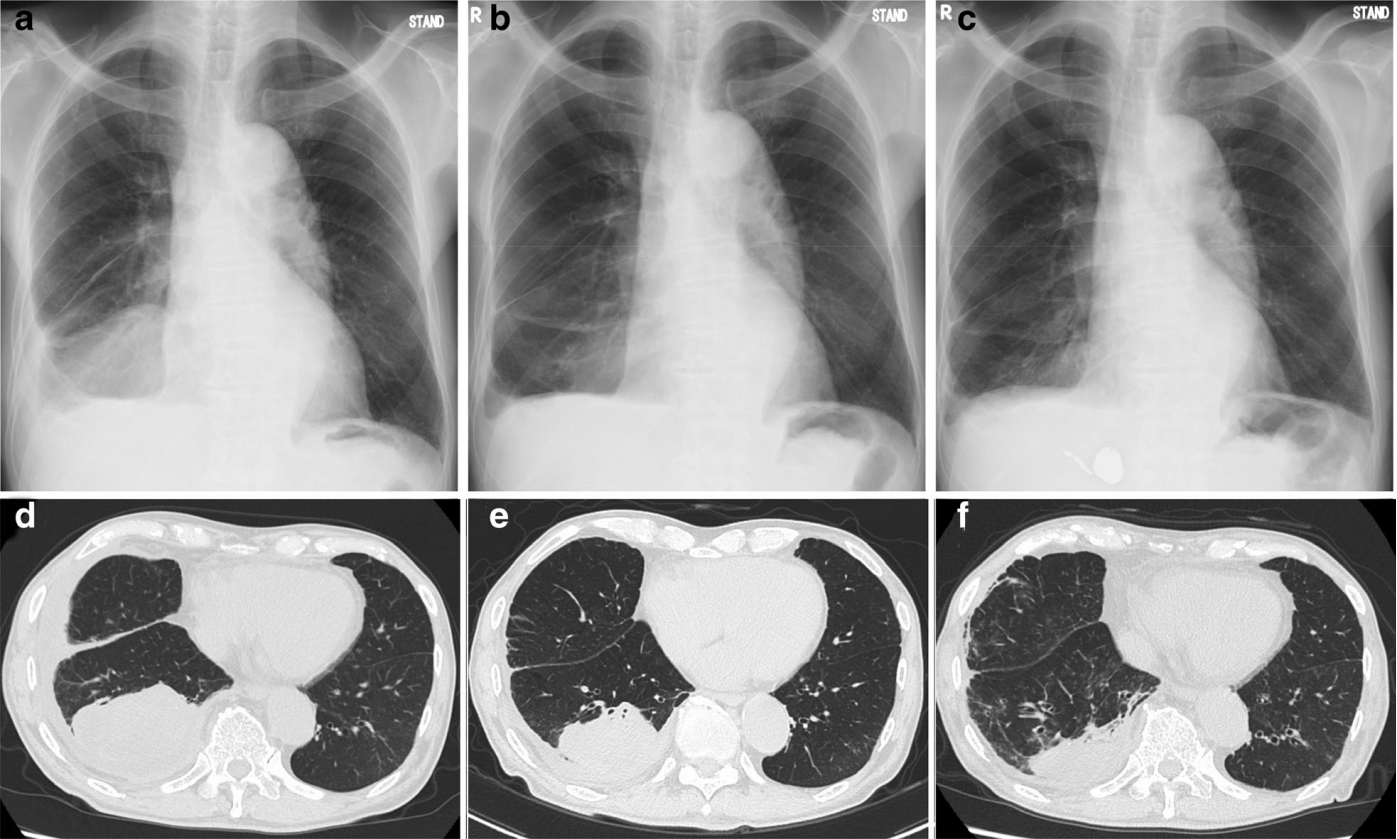
Chest X‐rays and computed tomography scans obtained (**a**) (**d**) before and (**b**) (**e**) two weeks and (**c**) (**f**) seven weeks after the start of the dabrafenib plus trametinib treatment.

An 86‐year‐old male patient with a history of smoking, one pack per day from the age of 20 to 70 years of age, was referred to our hospital with dyspnea. A computed tomography (CT) scan revealed a lesion in the right lower lobe together with pleural effusion (Fig 1a,d) and multiple mediastinal lymphadenopathy. Positron emission tomography/computed tomography (PET/CT) also showed increased metabolic activity in the bilateral adrenal glands. Radiographic and pathological evaluations resulted in a diagnosis of advanced lung adenocarcinoma (cT4N2M1c, stage IVB). Next‐generation sequencing (NGS) revealed a *BRAF* V600E mutation in exon 15 (c.1799>A). The tumor sample was negative for other driver mutations, and immunohistochemistry (clone 22C3) demonstrated high programmed death‐ligand 1 expression (TPS 90%). Laboratory analyses revealed the following: leukocyte count: 7100/μL, hemoglobin level: 11.5 g/dL, C‐reactive protein level: 1.56 mg/dL, total protein level: 8.6 g/dL, albumin level: 2.4 g/dL, blood urea nitrogen level: 18 mg/dL, serum creatinine level: 0.85 mg/dL, and albuminuria score: 1+. These laboratory findings were indicative of mild hypoalbuminemia. Although the patient was “oldest old”, he had an Eastern Cooperative Oncology Group performance status (PS) of 1 and was treated with dabrafenib and trametinib combination targeted therapy. Dabrafenib was administered orally at a dose of 150 mg twice daily, and 2 mg trametinib was administered orally once daily.

**Figure 2:**
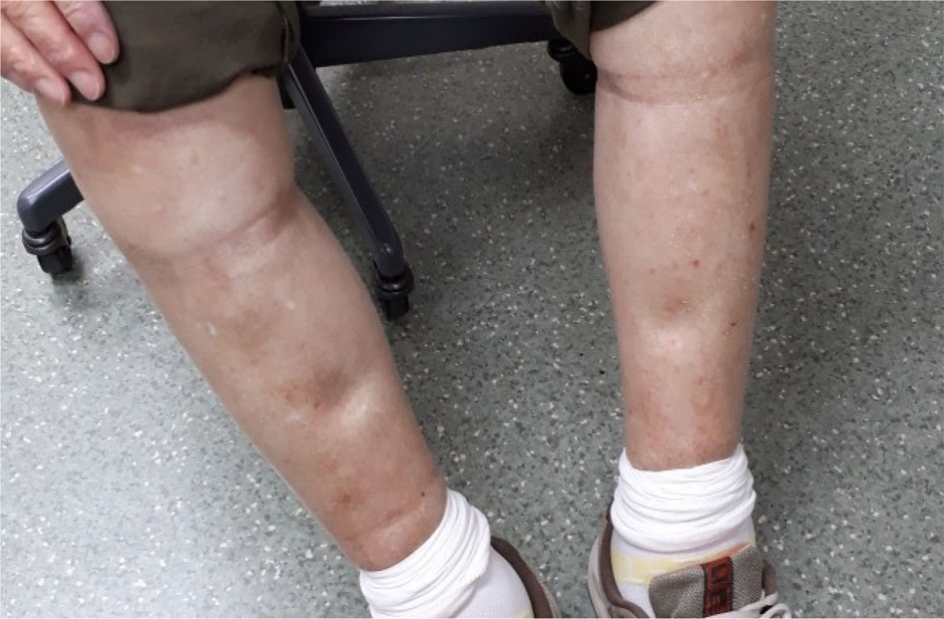
Peripheral pitting edema seen four weeks after the initiation of dabrafenib plus trametinib therapy.

**Figure 3:**
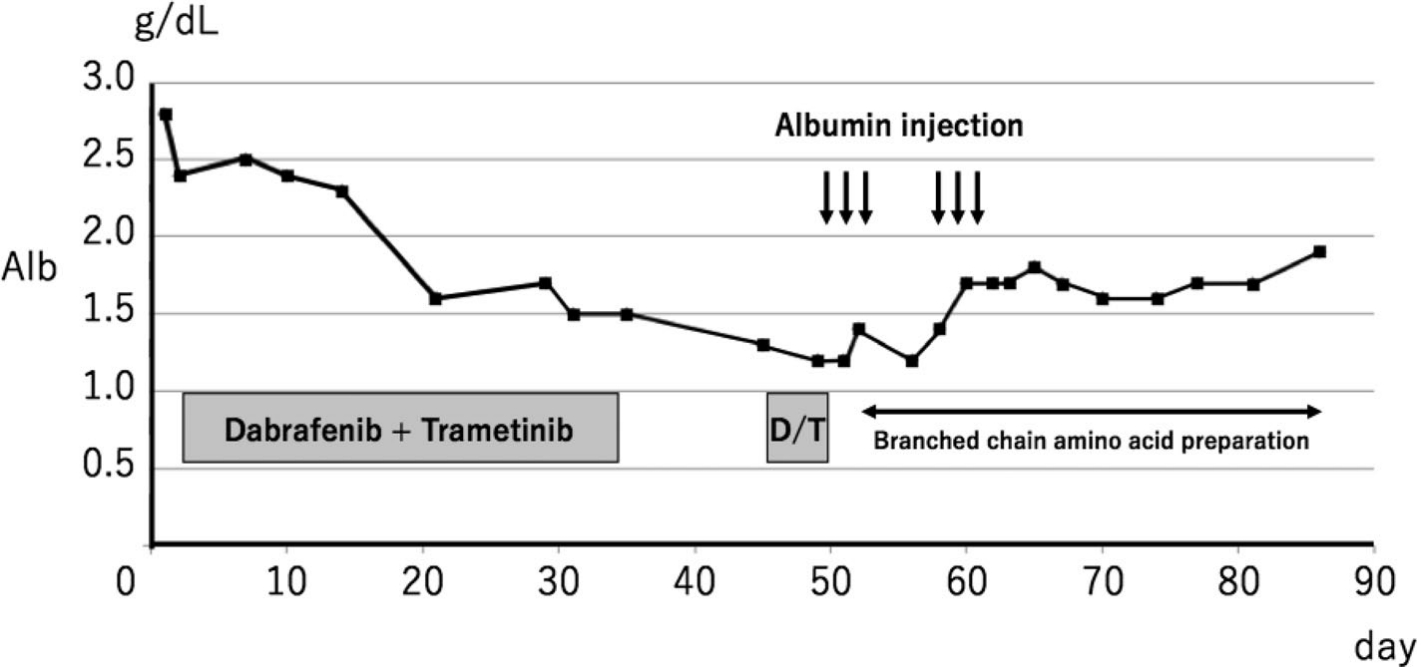
Timeline of the patient's serum albumin level. D/T: Dabrafenib plus trametinib.

After two weeks of targeted therapy, a chest X‐ray and CT scan revealed a reduction in the right pleural effusion and shrinkage of the primary mass (Fig 1b,e). During this period, the patient did not experience dyspnea, and his PS improved. Four weeks after the initiation of dabrafenib plus trametinib therapy, he developed peripheral leg edema and hypoalbuminemia (Fig 2). Ultrasound cardiography showed that his cardiac function had been preserved, and that he was not suffering from renal dysfunction, which suggested that the peripheral edema was related to the dabrafenib plus trametinib therapy. The peripheral edema created some problems for the patient during daily living activities and he was classified as grade 3 according to the Common Terminology Criteria for Adverse Events (CTCAE v5.0). The dabrafenib plus trametinib treatment was interrupted, and oral administration of the diuretic furosemide was initiated. The peripheral edema had not improved one week after the interruption of treatment, and so treatment was not recommenced. A CT scan performed seven weeks after the initiation of treatment revealed further tumor shrinkage; however, it also showed pneumonia that was either infection or drug‐induced, although the patient did not experience any symptoms. Thus, he was hospitalized and treated with intravenous antibiotics, albumin injections, and a branched‐chain amino acid preparation, and his pneumonia and hypoalbuminemia gradually improved. The blood albumin levels of the patient are shown in Figure 3. Dabrafenib and trametinib treatment was discontinued because of the risk of life‐threatening adverse events, and best supportive care was initiated. The duration of the dabrafenib and trametinib treatment was in total 40 days. Tumor shrinkage continued to be seen on radiological imaging (Fig 1c,f), and the patient was able to maintain his quality of life (QOL) for at least six weeks after treatment interruption.

## Discussion

This report describes the case of an “oldest old” patient with *BRAF* V600E‐mutated NSCLC, who received dabrafenib plus trametinib treatment. Although it induced a marked response, the patient was unable to continue to receive combination treatment because he developed grade 3 hypoalbuminemia and peripheral edema and was subsequently switched to supportive care. As targeted therapies for patients with NSCLC are usually expected to have marked therapeutic effects with only mild adverse events, even in elderly patients, this combination therapy might have caused relatively strong adverse events or the patient might have been too old. We consider that these observations will be helpful for clinicians facing similar situations in the future.

Targeted therapies have been previously reported to show marked antitumor effects against advanced NSCLC that express molecular driver oncogenes, such as epidermal growth factor receptor (EGFR), anaplastic lymphoma kinase (ALK), or c‐Ros oncogene 1 (ROS1),^5^, ^6^, ^7^, ^8^ including in elderly populations, resulting in the development of individualized treatment strategies. BRAF is a serine/threonine kinase, which is mediated by the RAS‐RAF‐MEK‐ERK signaling pathway,^1^ and *BRAF* mutations have been previously observed in about 1%–5% of lung adenocarcinomas.^2^, ^9^, ^10^ Targeting *BRAF* V600‐mutant tumors with selective BRAF and MEK inhibitors is the current standard treatment for patients with metastatic melanoma, in which the *BRAF* V600E mutation is more common than in NSCLC.^11^ Phase II trials of combined BRAF and MEK inhibition in patients with previously treated and untreated NSCLC achieved overall response rates of 63% and 64% and median response durations of 9.7 and 10.4 months, respectively.^3^, ^4^ Therefore, dabrafenib plus trametinib is the current standard treatment for patients with *BRAF* V600‐mutated advanced NSCLC, and was very appropriate for the present case.

There is limited clinical information about patients harboring *BRAF* mutations, especially in elderly patients and those with poor PS, because *BRAF* mutations are only found in a few populations. Intensive treatment, such as combination chemotherapy or combined modality, does not usually produce a survival benefit in patients aged >70 or 75 years.^12^, ^13^, ^14^ On the other hand, the use of therapies targeting other driver oncogenes is recommended, regardless of the patient's age and general condition, because of the marked responses they induce and their mild toxicities.^15^, ^16^, ^17^ However, the patient in the present case was 86 years old, which is classified as “oldest old”, and the supportive care rates in this age group range from 55%–67%.^18^, ^19^ Despite this, it seems that the treatment caused an improvement in QOL and prolonged the patient's life by shrinking the tumor, and thus, it had meaningful effects.

The most common adverse events of dabrafenib and trametinib therapy for NSCLC have been reported to be pyrexia, elevated alanine aminotransferase levels, hypertension, anemia, a confused state, decreased appetite, hemoptysis, hypercalcemia, nausea, vomiting, neutropenia, hyponatremia, and a reduction in the ejection fraction.^3^, ^4^ Previous trials have reported that 52% of melanoma patients that were treated with combination therapy had grade 3 or 4 side effects, and the frequencies of adverse events that led to dose reduction and treatment interruption were 33% and 55%, respectively.^20^ Therefore, clinicians must always keep in mind that the toxicities of combination therapy are relatively serious. In addition, the characteristics of the adverse events seen in patients treated with combination therapy often differ from those of other targeted therapies. Peripheral edema is a common adverse event and has been reported to occur in 28%–36% of cases of *BRAF* V600E‐mutant NSCLC,^3^, ^21^ but most patients experienced mild (grade 1 or 2) peripheral edema. To the best of our knowledge, there have been no reports about peripheral edema due to combination therapy that led to dose reduction or treatment interruption or discontinuance in NSCLC. However, in the present case the peripheral edema caused by hypoalbuminemia triggered a decline in the patient's QOL, which could be intolerable for some patients. In addition, when peripheral edema is observed, clinicians must always exclude a reduction in the left ventricular ejection fraction and cardiac tamponade using ultrasound cardiography.^22^ Liver dysfunction did not occur in the present case; thus, hypoalbuminemia was not derived from liver toxicity. As the ability to assimilate nutrients decreases, tumor‐induced inflammation increases, and the clearing of necrotic tumor cells from the body after durable treatment becomes less effective with age, and it might be necessary to monitor patients for chemotherapy‐induced hypoalbuminemia and peripheral edema when chemotherapy is administered to “oldest old” patients. Patients with bilateral adrenal metastases and adrenal insufficiency might also present with hypoalbuminemia. During the patient's clinical course, pneumonia also appeared for a period of time, and the possibility of drug‐induced pneumonitis was feared. The presence of fibrosis on chest CT before treatment has been reported to predict the appearance of anticancer drug‐induced pneumonia,^23^, ^24^ but this was not seen before treatment in the case reported here, and the patient's pneumonia improved with antibacterial treatment and drug suspension; therefore, drug‐induced pneumonia could not be completely ruled out with no retreatment. If the tumor had not possessed the *BRAF* V600E mutation, no other treatment strategies would have been available for the patient due to his age. Thus, we consider that the combination therapy administered in this patient had many benefits. A previous study reported that a reduced dose of combination therapy prevented toxicities and maintained antitumor effects^25^; therefore, dose reduction from the beginning of treatment might be a better option for elderly patients, as it might allow combination therapy to be continued successfully.

Immunohistochemistry (clone 22C3) demonstrated high programmed death‐ligand 1 expression (TPS 90%). Regarding the issue which is better about targeted drug or immune check point inhibitor for elderly patients with both BRAF mutation and high expression of PD‐L1 more than 90% TPS. It was considered that immunotherapy may be less effective for driver gene positive cancer and in elderly people; in addition, it has also been previously reported that there is a risk of increased drug‐induced pneumonia after immunotherapy there is a risk of drug‐induced pneumonia of EGFR‐TKI such as osimertinib.^26^ Therefore, we decided to first administer targeted therapy. The patient did not use immunotherapy after all, but if he was fine with a good PS, immunotherapy was considered the option after targeted therapy.

In conclusion, dabrafenib and trametinib caused marked tumor shrinkage, improvements in QOL, and prolonged survival when administered to an “oldest old” patient with *BRAF* V600E‐mutant NSCLC. Active targeted therapy may therefore be considered instead of conservative treatment even if the patient is extremely old. In such cases, careful management, including treatment interruption and dose reduction, is required to manage adverse events such as hypoalbuminemia and peripheral edema derived from the decline of the patient's physical condition,

## Disclosure

The authors report no conflicts of interest related to this work.
